# Biogeophysical and physiological processes drive movement patterns in a marine predator

**DOI:** 10.1186/s40462-017-0107-z

**Published:** 2017-07-18

**Authors:** Lucy A. Howey, Bradley M. Wetherbee, Emily R. Tolentino, Mahmood S. Shivji

**Affiliations:** 1 Save Our Seas Shark Research Center and Guy Harvey Research Institute, Nova Southeastern University, Fort Lauderdale, Florida, USA; 2 Department of Biology, University of Rhode Island, Kingston, RI USA; 3 Microwave Telemetry, Inc., 8835 Columbia 100 Parkway, Suites K & L, Columbia, MD USA

**Keywords:** Blue shark, Pelagic, Sexual segregation, Migration, Movement, Satellite tracking, Atlantic Ocean, Vertical behavior, Aggregation

## Abstract

**Background:**

Blue sharks (*Prionace glauca*) are among the most abundant and widely distributed of oceanic elasmobranchs. Millions are taken annually in pelagic longline fisheries and comprise the highest component of auctioned fin weight in the international shark fin trade. Though studies of blue sharks outnumber those of other large pelagic sharks, the species’ complicated and sexually segregated life history still confound current understanding of Atlantic movement patterns. Lack of detailed information regarding movement and vertical behavior continues to limit management efforts that require such data for stock assessment and sustainable catch modeling. Therefore, this study aims to describe behavioral and ecological patterns distinct to aggregating and migrating blue sharks, and compare the findings to existing Atlantic movement models.

**Results:**

Data collected from 23 blue sharks instrumented with pop-up satellite archival tags were used in statistical predictive regression models to investigate habitat use during a localized aggregation in the northwest Atlantic, while undergoing seasonal migrations, and with respect to environmental variables. Deployment durations ranged from 4 to 273 days, with sharks inhabiting both productive coastal waters and the open ocean, and exhibiting long-distance seasonal movements exceeding 3700 km. While aggregating on the continental shelf of the northwest Atlantic, blue sharks displayed consistent depth use independent of sex and life stage, and exhibited varied response to environmental (temperature and chlorophyll *a*) factors. As sharks dispersed from the aggregation site, depth use was influenced by bathymetry, latitude, demography, and presence in the Gulf Stream. Mature females were not observed at the New England tagging site, however, two mature females with recent mating wounds were captured and tagged opportunistically in The Bahamas, one of which migrated to the Mid-Atlantic Ridge.

**Conclusions:**

Vertical behaviors displayed by blue sharks varied greatly among locales; depth use off the continental shelf was significantly greater, and individuals exhibited a greater frequency of deep-diving behavior, compared to periods of aggregation on the continental shelf. Sexual segregation was evident, suggesting mature and immature males, and immature females may be subjected to high levels of anthropogenic exploitation in this region during periods of aggregation. Analysis of the spatio-temporal tracks revealed that nine individuals traveled beyond the United States EEZ, including a mature female captured in The Bahamas that migrated to the Mid-Atlantic Ridge. These results reflect and augment existing Atlantic migration models, and highlight the complex, synergistic nature of factors affecting blue shark ecology and the need for a cooperative management approach in the North Atlantic.

**Electronic supplementary material:**

The online version of this article (doi:10.1186/s40462-017-0107-z) contains supplementary material, which is available to authorized users.

## Background

As a central component of animal ecology, movement enables animals to respond to their environment, increase growth and survival, optimize foraging efforts, and increase reproductive success [1]. The operational scale and pattern of marine animal movement varies widely among taxa, and understanding the relationship between a species and its environment is vital for effective conservation and management [2–5]. Since apex predators often provide structural integrity for marine food webs, and exhibit naturally slow population growth, fisheries regulation and protection of elasmobranchs and large teleosts have become high priority in the last decade [6–10]. Multilateral management of animals that migrate across political boundaries requires international cooperation and an accurate description of transboundary and shifting spatial use [11, 12].

Blue sharks (*Prionace glauca*) are ectothermic, large-bodied elasmobranchs, common in temperate and tropical waters, and exhibit changes in spatial distribution through wide-ranging and complex annual migrations [13–15]. Increasing catch rates [16] and a high prevalence in the international fin trade [17, 18] are potential causes of population declines [19, 20] and further the need for global management [21]. Effective management of blue sharks, in-part, hinges on identifying stocks and characterizing population structure based on migratory patterns, habitat use, and the differences displayed by demographic group when quantifying such behaviors.

Catch records and genetic evidence suggest distinct northern and southern Atlantic stocks of blue sharks [21–23], which exhibit limited movement across equatorial boundaries [14, 22, 24]. A 40-year National Marine Fisheries Service (NMFS) Apex Predator Program mark-recapture study of >100,000 blue sharks, many of which were tagged off the coast of New England during seasonal abundances [24], has served as the basis of a North Atlantic movement model [22, 25, 26]. In the western Atlantic, mature male and immature blue sharks (of both sexes) are commonly observed between May and October on the US North Atlantic continental shelf. This coincides with the New England recreational fishing season and may subsequently result in the increased targeting of blue sharks [27]. The aggregation’s proximity to land and shallow continental shelf waters, which range from 60 to 200 m depth at the shelf edge [28–30], is atypical of pelagic and epipelagic sharks [31] suggesting that this area is of life-history importance. Blue shark prey, which consists primarily of cephalopods and teleosts, is locally abundant during this time [32], suggesting that seasonal occurrence of blue sharks is related to feeding [22]. The incidence of mating wounds and presence of sperm in the less commonly observed females (both mature and immature) suggests that some mating occurs during this summer aggregation [22, 24, 33]. In the late summer, these sharks embark on migrations, traveling as far south as the Caribbean and South America [15, 25]. Sub-adult females may ride current systems (i.e., Gulf Stream) to the eastern Atlantic [25]. Sexual segregation is evident across the Atlantic basin and data modeling suggests that mating in the northern hemisphere occurs in the spring and summer [25]. Blue shark literature suggests a yearly reproductive cycle and gravid females are common in the winter near the Canary Islands and the north African coast [25, 33, 34]. Parturition occurs during early spring in Mediterranean nursery grounds and off the Iberian Peninsula, and particularly near the Azores [26, 35, 36]. Poleward migrations of sexually segregated populations occur during spring and summer and complete the annual circuit.

Accurate description of vertical behavior is an important component in migration modeling. The ability to describe, not only where an animal moves, but how it moves, is especially important for sexually segregated species that are exposed to fluctuating abiotic and biotic variables while moving long distances. Several acoustic and active tracking studies of Atlantic blue sharks have yielded fine-scale vertical movement and behavioral data that provide insight into post-release recovery, depth use, and physiology [37, 38]. In both the Atlantic and Pacific, blue sharks routinely demonstrate oscillatory dives between the surface and 400 m and exhibit diel variation in depth. These behaviors are often ascribed to hunting and behavioral thermoregulation [37, 39]. However, existing Atlantic movement models of blue sharks have omitted vertical components because advances in technology have only recently made this possible.

Satellite tracking technology has become a key component in answering habitat-related questions associated with migratory marine animals that occupy large niches, such as whales [4, 5], turtles [40], teleosts [41–45], and elasmobranchs [31, 46, 47] including blue sharks [15]. In this study, pop-up satellite archival tags (hereafter referred to as PSATs) providing depth, temperature and light-based geolocation data, enabled empirical testing of several hypotheses: 1) high abundance of blue sharks on the US continental shelf during summer months supports a physiological process (i.e., reproduction or feeding), 2) long-term movements observed via sequentially point-measured satellite tracking in the northwest Atlantic align with, and extend existing migration models that are based on mark-recapture data, and 3) demographic groups common in the western Atlantic exhibit distinct habitat use that is directly dependent on (a) biotic variables (e.g., chlorophyll, SST, bathymetry, ambient pressure[depth] and temperature).

## Methods

### Shark capture and handling

Blue shark tagging occurred at three separate tagging sites. Near Martha’s Vineyard, MA, USA, sharks were captured on rod and reel using Atlantic mackerel (*Scomber scombrus*) or bluefish (*Pomatomus saltatrix*) baited circle hooks from sport fishing boats (Table 1). Once captured, a rope was placed around the caudal fin and the shark was suspended in the water alongside the vessel for tagging, length measurement and determination of sex and maturity. Sharks were tagged with a NMFS Apex Predator program M dart tag [14] and with a PSAT tag (Standard Archival or X-Tag, Microwave Telemetry, Inc., Columbia, MD) anchored into the dorsal musculature on opposing sides of the dorsal fin. PSATs were anchored with plastic umbrella darts [48] with 20 cm of 220-lb test monofilament encased in surgical silicone tubing. Combined angling and handling time averaged 22.5 min (SE ± 2.61) and hooks were removed prior to release. A small tissue sample was taken from each shark for a genetics study and to provide a genetic fingerprint of each individual. Release location, time, water temperature and bottom depth were recorded for each tagged shark.

**Table 1:** Biological information for animals instrumented in the study

ID	Deployment date	Tag type	Tagging site	Deployment location	End date	End location	Sex	Stage of maturity	Fork length (cm)	Habitat relative to continental shelf
43908 (S1)	6/16/2007	Archival HR	NW Atlantic Shelf	40.86 °N, 71.23 °W	7/1/2007	40.82 °N, 71.61 °W	M	IM	214	Excluded^a^
43915 (S2)	6/18/2007	Archival HR	NW Atlantic Shelf	40.90 °N, 70.86 °W	6/22/2007	39.59 °N, 71.96 °W	M	IM	209	Excluded^a^
43953 (S3)	6/19/2007	Archival SR	NW Atlantic Shelf	40.93 °N, 70.86 °W	8/1/2007	40.86 °N, 66.82 °W	M	IM	212	On/Off
43954 (S4)	6/19/2007	Archival SR	NW Atlantic Shelf	40.94 °N, 70.89 °W	7/8/2007	40.82 °N, 71.61 °W	M	M	235	On
43963 (S5)	6/17/2007	Archival SR	NW Atlantic Shelf	40.90 °N, 70.84 °W	6/23/2007	40.74 °N, 71.15 °W	F	IM	171	On/Off
43968 (S6)	8/15/2007	Archival SR	NW Atlantic Shelf	41.08 °N, 70.81 °W	9/12/2007	40.29 °N, 70.70 °W	M	IM	184	On/Off
43973 (S7)	6/17/2007	Archival SR	NW Atlantic Shelf	40.90 °N, 70.88 °W	6/23/2007	41.71 °N, 68.85 °W	F	IM	154	On/Off
43984 (S8)	8/13/2007	Archival SR	NW Atlantic Shelf	41.04 °N, 70.62 °W	2/13/2008	31.00 °N, 59.15 °W	M	M	241	On/Off
43985 (S9)	9/14/2007	X-Tag SR	NW Atlantic Shelf	40.93 °N, 70.99 °W	10/22/2007	36.47 °N, 64.47 °W	M	M	246	On/Off
43987 (S10)	9/14/2007	X-Tag SR	NW Atlantic Shelf	40.88 °N, 70.93 °W	12/5/2007	38.12 °N, 68.76 °W	M	IM	188	On/Off
44004 (S11)	9/13/2007	X-Tag SR	NW Atlantic Shelf	40.85 °N, 70.92 °W	9/25/2007	39.22 °N, 67.63 °W	M	IM	218	On/Off
44014 (S12)	8/13/2007	Archival SR	NW Atlantic Shelf	41.04 °N, 70.58 °W	2/13/2008	18.73 °N, 67.34 °W	M	M	297	On/Off
44047 (S13)	9/13/2007	X-Tag SR	NW Atlantic Shelf	40.86 °N, 70.92 °W	4/13/2008	38.38 °N, 66.45 °W	M	IM	211	On/Off
44061 (S14)	9/13/2007	X-Tag SR	NW Atlantic Shelf	40.86 °N, 70.92 °W	10/10/2007	36.89 °N, 69.26 °W	M	M	249	On/Off
85894 (S15)	10/12/2008	Archival SR	NW Atlantic Shelf	42.09 °N, 70.31 °W	7/12/2009	26.55 °N, 73.32 °W	F	IM	170	On/Off
85895 (S16)	4/26/2009	Archival SR	Sargasso Sea	26.45 °N, 70.07 °W	8/31/2009	31.06 °N, 57.72 °W	F	IM	163	Off
85900 (S17)	8/30/2008	X-Tag SR	NW Atlantic Shelf	40.89 °N, 70.89 °W	10/30/2008	39.14 °N, 71.72 °W	M	IM	172	On/Off
85902 (S18)	8/31/2008	Archival SR	NW Atlantic Shelf	41.03 °N, 70.65 °W	11/10/2008	37.90 °N, 70.44 °W	M	IM	176	On/Off
85903 (S19)	9/1/2008	Archival SR	NW Atlantic Shelf	41.08 °N, 70.84 °W	3/1/2009	37.19 °N, 74.81 °W	F	IM	167	On/Off
115974 (S20)	5/19/2015	X-Tag SR (Recovered)	The Bahamas	24.13 °N, 75.33 °W	11/4/2015	32.80 °N, 38.60 °W	F	M	215	Off^b^

Stage of maturity denoted as IM (immature) and M (mature)

^a^Excluded from all habitat (on-shelf versus off-shelf) analyses since High Rate tags do not include location estimation (and thus presence on/off continental shelf could not be confirmed)

^b^Excluded from linear mixed effects models since this individual was the only mature female, but included in off-shelf summary statistics

Additional blue sharks were opportunistically captured and tagged with PSATs by NMFS observers aboard longline vessels in the southern north Atlantic, and during an unrelated study in Cat Island, The Bahamas (Table 1). In The Bahamas, animals were captured on baited hand lines [31], and research was conducted under the Cape Eleuthera Institute (CEI) research permit (MAF/FIS/17 & MAF/FIS/34) issued by the Bahamian Department of Marine Resources.

### Satellite tag details

Standard Rate (SR) and High Rate (HR) X-Tags and Standard Archival Tags (Microwave Telemetry, Inc., Columbia, MD, USA) were used for this study. X-Tags were 12 × 3.2 cm (excluding antenna) and weighed 40 g in air. Standard Archival Tags were 16.6 × 4.1 cm (excluding antenna) and weighed 65 g in air. At a pre-programmed pop-up date (between 30 and 273 days for this study), the release mechanism corroded the link allowing the tag to detach from its tether and float to the surface to transmit data through the Argos satellite system. Tags were also programmed to detach under conditions of constant depth or after reaching a depth where the physical integrity of the tag may be compromised. Depending on the length of deployment, SR tags provide 15–60 min interval time-series depth and temperature records, and in the event that a SR tag was recovered, 2-min records could be extracted. Tags implement data compression techniques prior to transmission, and as a result, selected depth and temperature values in transmitted SR datasets may be identified as delta limited and not represent the full extent of vertical or temperature range [49]. Specifically, the depth of the shark corresponding to a depth record marked by a delta limited dive or ascent and may have actually represented deeper or shallower depths than indicated. Delta limited temperature records followed a comparable pattern. Nonetheless, all delta limited values were included in the analyses. Additionally, the SR tag’s onboard algorithm calculates daily sunrise and sunset times from 2-min recorded light levels. Daily geolocations are subsequently calculated from the transmitted daily sunrise and sunset times (error at best ±1° latitude and ±0.5° longitude). HR tags, programmed for 30-day deployments, provided time-series depth and temperature records at approximately five-minute intervals but do not provide location estimates. Because of battery and Argos system throughput limitations, only a subset of transmitted records may be received through the satellite system. From the tags used in this study, transmitted SR depth resolution is 5.4 m, and recovered SR depth resolution was 0.34 m. HR depth resolution was 1.34 m. The temperature resolution for all tags (SR and HR) ranged between 0.16 to 0.23 °C [50].

### Data treatment

All available geolocations from SR tags were processed with a state-space unscented Kalman filter with sea surface temperature (UKFSST) [50]. Bounds were initially applied to tracks (Longitude bounds: 30 °W to 100 °W, Latitude bounds: Equator to 60 °N) to remove erroneous location estimates prior to processing. The NOAA Optimum Interpolation Sea Surface Temperature V2 dataset served as the weekly, one-degree SST field [51, 52] and daily maximum temperature records provided daily SST estimates. For days without a transmitted maximum temperature, local polynomial smoothing was applied to approximate missing values [53]. After UKFSST processing, a bathymetric correction was applied to create the final estimated track and remove erroneous location estimations, such as those on land [54, 55]. The UKFSST estimated positions combined with the corresponding variance parameters were used to construct utilization distributions [53]. All location analyses were completed with the “analyzepsat” package in R [53–56].

To visualize the on-shelf spatial distribution with respect to primary production, chlorophyll a concentration data were obtained from the NOAA CoastWatch dataset Chlorophyll *a* Aqua Modis Global Science Quality (Monthly Composite) [57]. In order to combine data over three years of the study, the chlorophyll concentrations for corresponding months were averaged from the study years (2007–2009). Due to the exponential distribution of chlorophyll concentrations, data were initially transformed (log_10_[x + 1]) to evaluate the arithmetic mean, and mean values were subsequently back-transformed.

Depth and temperature records from the first and last days of deployment were removed prior to the analysis to ensure the evaluation of natural behavior. UKFSST tracks provided information regarding position on or off the continental shelf based on the 200 m threshold [29]. For each UKFSST estimated position, bottom depth was retrieved from the one-minute (Lat/Long) resolution ETOPO1 Global Relief bathymetry dataset [55]. If bottom depth surpassed the approximate continental shelf edge depth of 200 m, the location (and the corresponding day’s depth and temperature data) was identified as being off the shelf. The remaining data were classified as located on the shelf. Next, all data were classified based on presence in the Gulf Stream, which was defined as area encompassed by the 20 °C SST contour. If daily maximum temperature was greater than 20 °C, then that day’s corresponding data (depth and temperature) records were considered to be in the Gulf Stream [58]. The remaining data were considered outside of the Gulf Stream. This temperature threshold method was more reliable than location based methods, due to the inherent error in light-based geolocations and the abrupt SST gradients in the North Atlantic. From the daily UKFSST estimated positions, corresponding sunrise and sunset times were calculated. Crepuscular periods, dawn and dusk, were assigned to data that fell within one hour before sunrise or after sunset. Remaining data were designated day or night. Additionally, a daily distance metric (measured in km/day) was calculated for each individual that exhibited movement across the 40th parallel north (40 °N) (*n* = 9). To test the null hypothesis that level of relative dispersal by an individual in spatially delineated focal areas does not differ, a two-sample t-test was performed with a sample from productive waters (≤40 °N) and a sample from an area with lower primary productivity (≥40 °N).

### Statistical analyses

R 2.15.3 was used for all analyses [59]. A significance level of 0.05 was used in all statistical analyses, and Spearman’s rank was used for reported correlations. To evaluate vertical behavior and the concomitant temperature selection across environments encountered in this study, linear mixed effects models were constructed to specifically investigate how depth and temperature use varied among habitats and demographic groups (see Additional file 1). Only data from SR tags were considered because HR tags do not provide the necessary location estimates required for determination of location relative to the shelf (Table 1). Additionally, in comparing demographic groups, we excluded the single mature female dataset (Table 1). In all models, a random effect was modeled for each individual shark to account for repeated observations from each individual. The first model considered the response variables of daily mean depth as a function of the interaction between the continental shelf (marked by a 200 m bathymetry threshold) and Gulf Stream (identified by 20 °C SST threshold) factors (Table 2). In subsequent models, data were partitioned based on location relative to the continental shelf habitat. For both the on-shelf and off-shelf habitats, day and night mean depth and temperature (for each day) were independently modeled as the response to the additive effect of diel period (day and night), demographic group (immature females, immature males, and mature males), and Gulf Stream (inside or outside) (Table 2). Response variables were Box-Cox transformed prior to analysis. All models were assessed for violations to model assumptions. Issues of unequal variance between factor levels were resolved by adding a heteroscedastic variance structure to the models. Issues of residual correlation, identified with an autocorrelation function plot, were accounted for with an autoregressive moving average (ARMA) correlation structure [60], using the “nlme” package in R [61]. For model results indicating a significant factor, simultaneous tests for general linear hypotheses with Tukey contrasts were applied with the “multcomp” package in R [62, 63].

**Table 2:** Summary of linear mixed effect models. See Additional file 1 for additional details

Model	Habitat	Response variable	Fixed effects	Correlation structure	Variance structure
1	All habitats	Daily mean depth	Shelf*Gulf	X	X
2	On-shelf habitat	Daily day/night mean depth	Period + Demographic Group + Gulf Stream		X
		Daily day/night mean temperature	Period + Demographic Group + Gulf Stream	X	X
3	Off-shelf habitat	Daily day/night mean depth	Period + Demographic Group + Gulf Stream	X	X
		Daily day/night mean temperature	Period + Demographic Group + Gulf Stream	X	

## Results

**Fig. 1:**
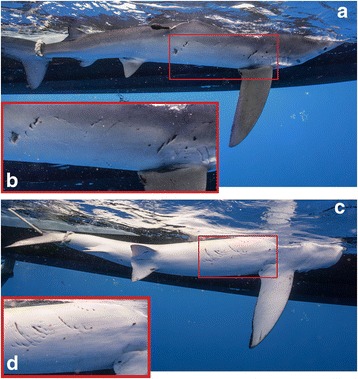
Recent (estimated to have occurred within 2 weeks prior to capture) mating wounds observed on mature female (115974, S20) in The Bahamas. Image (**a**) is associated with zoomed-in inset (**b**), and image (**c**) is associated with inset (**d**). Photos are by Andy Mann

A total of 33 tags was deployed on blue sharks between June 2007 and May 2015 (16 Standard Archival Pop-up Tags, 2 High Rate Archival Pop-up Tags, and 15 X-Tags). Most tags (*n* = 29) were deployed in the northwest Atlantic (24 males, 5 females) on the continental shelf, south of Martha’s Vineyard, MA, USA. Two tags were deployed in the southern North Atlantic (one male and one female). The remaining two tags were deployed on adult females near Cat Island, The Bahamas. Both individuals had noticeable mating wounds, however the larger mature female had deep, and presumably fresh (estimated ≤2 weeks, personal observation), mating wounds (Fig. 1).

Data were retrieved from 23 tags (69.7%). However, three individuals appear to have died based on flat depth profiles following release and were omitted from all analyses. After two months at liberty, tag 85900 (deployed on immature male, S17) was most likely consumed by a predator, evidenced by an elevated temperature range and lack of recorded light levels. Therefore, only data from the period prior to suspected consumption were analyzed. The mature female (115974, S20) tagged in The Bahamas was captured by a European longlining vessel (32.8 °N, 38.6 °W); its tag was recovered and a complete archived dataset was retrieved. This individual was excluded from the statistical models pertaining to demographic group, but retained for general vertical and horizontal movement analyses.

The 20 useable datasets provided information on 9 immature males, 5 mature males, 5 immature females, and 1 mature female (Table 1). Percentage of archived data received by satellites ranged between 38 and 100% (mean ± SD = 80.9 ± 20.2%) and deployment durations averaged 86.9 ± 83.8 days (range: 4–273 days) (Table 1). Tags provided a total of 203,489 depth records and 204,161 temperature records; however, the majority of these records were from recovered X-Tag 115974 (121,557 depth records; 121,557 temperature records). Delta limited temperature values comprised 5.6% of the total transmitted temperature dataset comprised of all individuals (51.3% delta limited increases and 48.7% delta limited decreases), and delta limited depth values comprised 4.6% of the collective transmitted depth dataset from all sharks (53.3% delta limited dives and 46.7% delta limited ascents). Temperatures ranged between 3.9 and 31.3 °C; the coldest temperature was recorded by a mature male (43984, S8). Daily maximum tag-recorded temperatures, serving as a proxy for SST, ranged between 12.1 and 31.3 °C. The deepest depth recorded was 1291.1 m (± 5.14 m) by a mature male (44014, S12).

Daily depth range positively correlated with SST (*r*_*s*_ = 0.52, *P* < 2.2 × 10^−16^, *n* = 20) and daily temperature range positively correlated with SST (*r*_*s*_ = 0.71, *P* < 2.2 × 10^−16^, *n* = 20), so that higher surface temperatures corresponded to larger variations in daily depth and temperature. Daily maximum depth negatively correlated with latitude (*r*_*s*_ = −0.81, *P* < 2.2 × 10^−16^, *n* = 18), indicating that daily maximum depths increased as individuals traveled south to lower latitudes.

A significant interaction between the continental shelf and warm surface waters (SST >20 °C, indicating the Gulf Stream) predicted daily mean depth (*F*_1,1340_ = 13.76, *P* = 2 × 10^−4^). Post hoc analysis indicated that, independent of residence in warm waters (Gulf Stream, SST >20 °C), individuals used deeper waters off the continental shelf than on the shelf. On the shelf, presence in the Gulf Stream was not associated with mean depth differences. In contrast, off the shelf, the warm waters of the Gulf Stream were associated with increased depth, with a deeper mean depth associated with being inside the Gulf Stream compared to outside the Gulf Stream.

### On-shelf habitat use

**Fig. 2:**
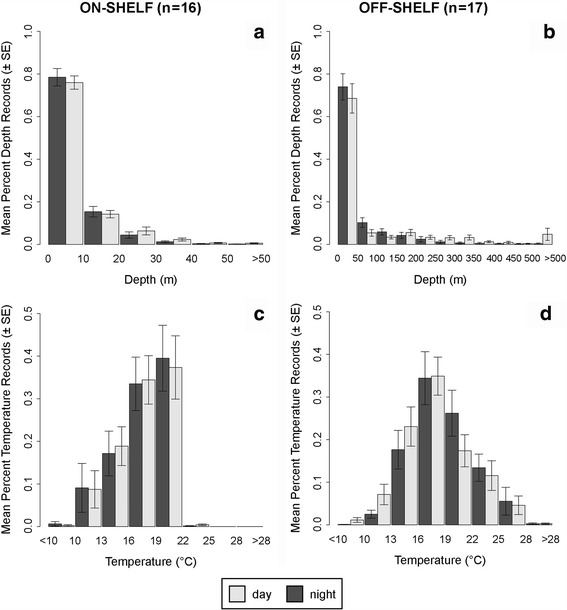
Percent time at depth (**a**–**b**) and temperature (**c**–**d**) on (left) and off (right) the continental shelf (marked by 200 m bathymetry contour) and separated by day and night

**Fig. 3:**
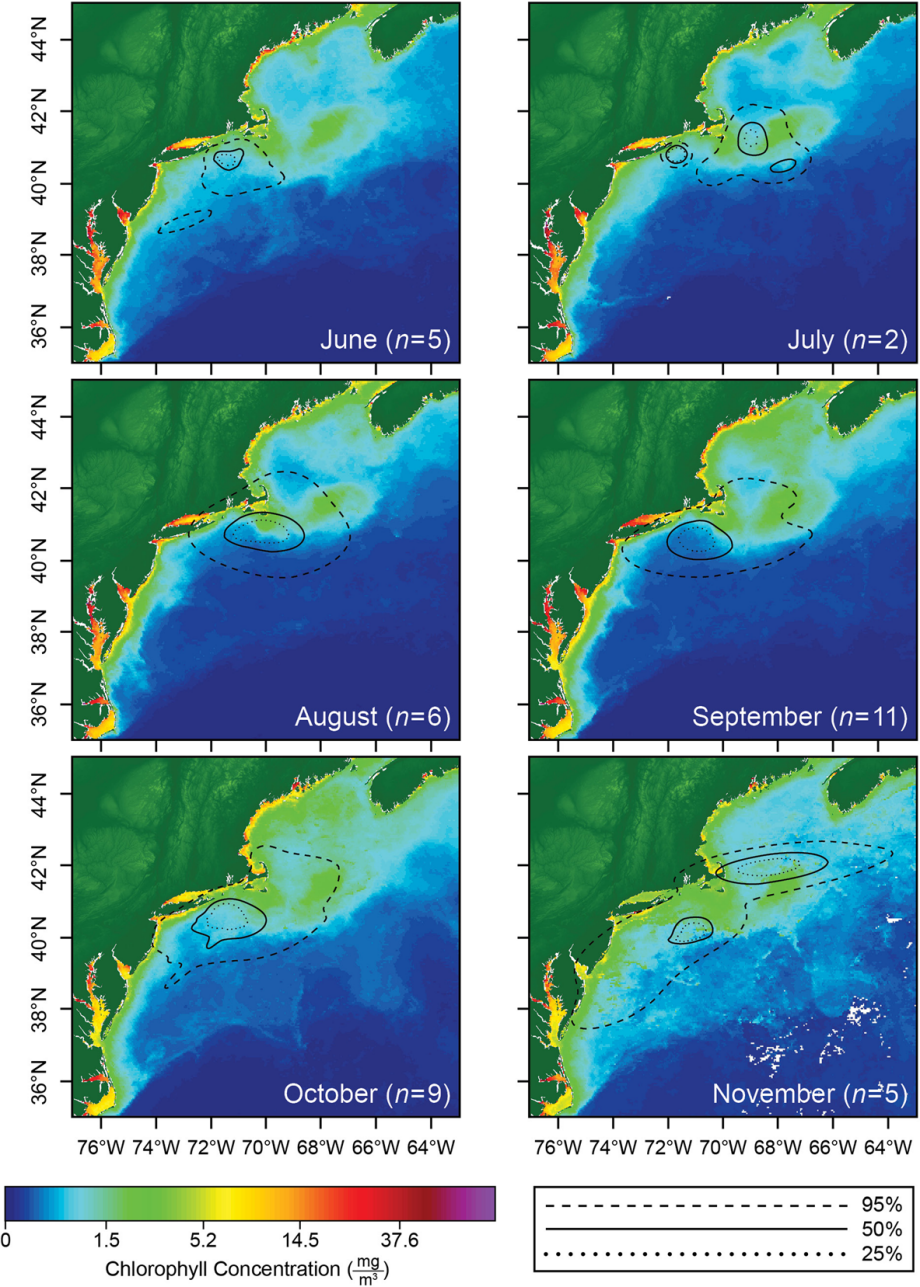
On-shelf utilization distribution contours representing the high density regions, derived from daily estimated locations, and considering only locations from the on-shelf habitat (marked by the 200 m bathymetry contour). Each month including blue shark shelf residency is displayed and overlaid on chlorophyll* a* concentration, indicative of primary productivity

Of the tag-derived UKFSST positions available, 427 locations (40.7%) from 16 individuals (88.9% of considered individuals) occurred on the continental shelf (<200 m) between the months of May and November. Of these locations (*n* = 190), 44.5% were in waters with SST >20 °C. Individuals spent an average of 44.7 ± 36.0% of time in waters with SST >20 °C (range: 0–100%). SSTs from on-shelf positions ranged between 12.1 °C and 23.1 °C and averaged 19.2 ± 2.2 °C. Sharks spent 72.0 ± 32.7% of time between temperatures 16–22 °C (Fig. 2). On the shelf, the mean maximum daily depth was 45.9 ± 25.8 m. The on-shelf 95% utilization distribution contour varied in size between months, ranging between 390.60 km^2^ (June) to 2158.37 km^2^ (November) (Fig. 3). Correspondingly, daily linear movement (km/day) away from the aggregation site was significantly lower than daily movements in designated less productive regions (*t* = 1.20, *P* = 0.128).

In the model predicting on-shelf day/night mean depth, none of the factors considered were significant predictors of depth (Period: *F*_1,890_ = 0.003, *P* = 0.9579; Demographic Group: *F*_2,13_ = 1.34, *P* = 0.2945; Gulf Stream: *F*_1,890_ = 0.11, *P* = 0.7386). In contrast, demographic group and the Gulf Stream were significant factors predicting on-shelf day/night mean temperature (Demographic Group: *F*_2,13_ = 8.91, *P* = 0.0037; Gulf Stream: *F*_1,838_ = 237.52, *P* = <0.0001). However, diel period was not a significant factor (Period: *F*_1,838_ = 3.18, *P* = 0.0750). Specifically, mean temperatures were colder outside of the Gulf Stream compared to within the Gulf Stream. Immature females used lower mean temperatures than the mature males and immature males. No other demographic differences were identified.

### Off-shelf habitat use

Using the tag-derived UKFSST positions available, 786 locations from 17 individuals (94.4% of individuals) were registered off the continental shelf (bottom depth > 200 m), and 71.2% of these locations (*n* = 560) were in waters with SST >20 °C. Individuals spent an average of 58.8 ± 43.9% tracked time in waters with SST >20 °C (range: 0–100%). Off-shelf positions were recorded during all months and seasons of the year. SSTs from off-shelf positions ranged between 12.3 °C and 31.3 °C and averaged 22.56 ± 4.4 °C. Individuals spent 56.5 ± 20.6% of time between temperatures 16–22 °C (Fig. 2). Off the shelf, the mean maximum daily depth was 317.7 ± 192.9 m.

In the model predicting off-shelf daily day/night mean depth, all of the factors considered were significant predictors of depth (Period: *F*_1,1794_ = 60.96, *P* = <0.0001; Demographic Group: *F*_2,13_ = 5.48, *P* = 0.0188; Gulf Stream: *F*_1,1794_ = 28.90619, *P* < 0.0001). Specifically, post hoc analysis indicated that individuals were deeper within the Gulf Stream than outside the Gulf Stream and deeper during the day than night. Additionally, mean depth of mature males was greater than that of immature males. No other demographic differences were identified.

For the model predicting off-shelf daily day/night mean temperature, both diel period and Gulf Stream factors were significant (Period: *F*_1,1618_ = 21.35, *P* = <0.0001; Demographic Group: *F*_2,13_ = 0.54, *P* = 0.5948; Gulf Stream: *F*_1,1618_ = 149.76, *P* < 0.0001). Specifically, warmer temperatures were recorded during the night compared to day, and warmer mean temperatures were registered in the Gulf Stream compared to outside of the Gulf Stream.

### Mature female 115974

**Fig. 4:**
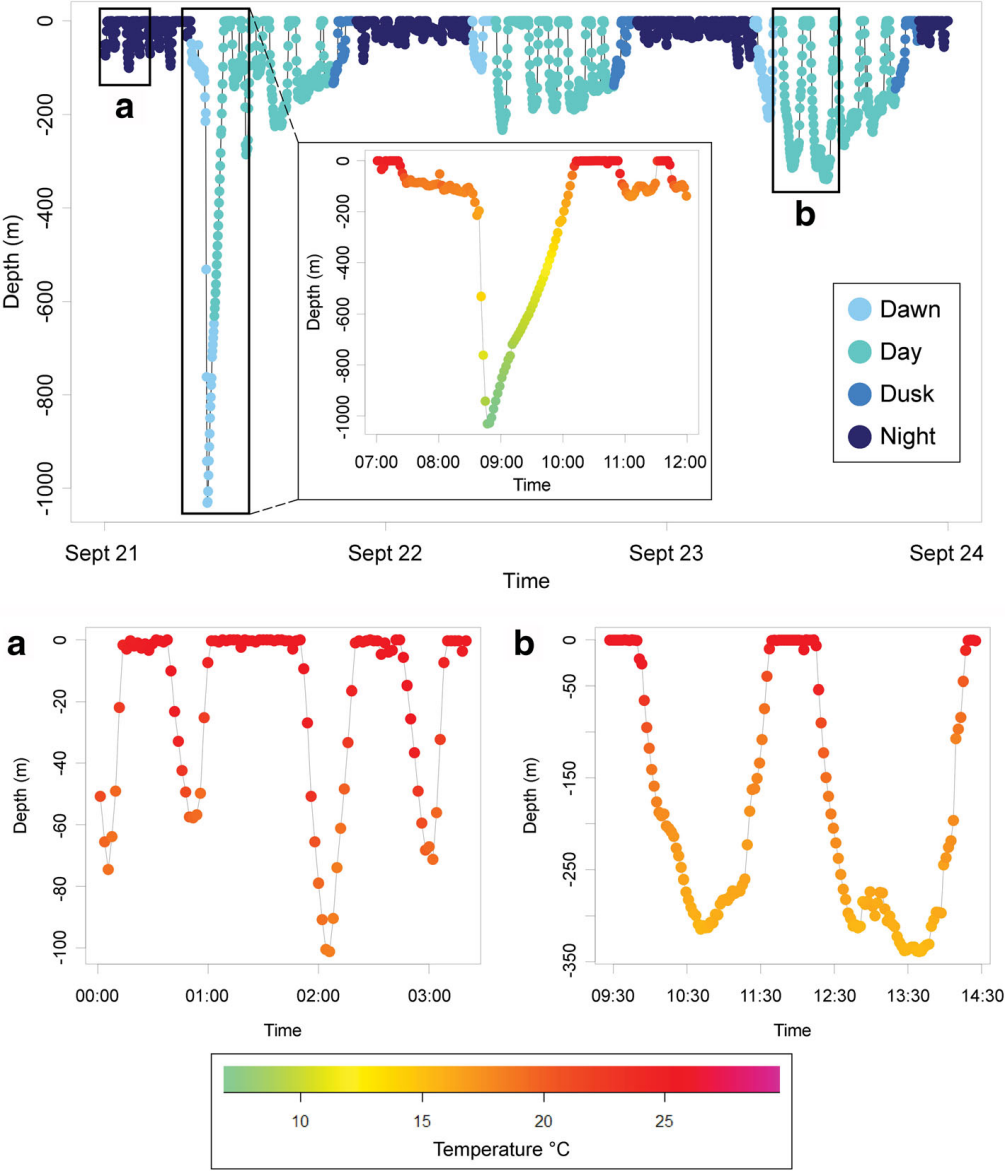
Segment of high-resolution (2-min) time-series depth data from the mature female (recovered X-Tag 115974, S20) colored by diel period to display diel variations, particularly deeper during the day. The three insets (two insets are labeled **a**–**b**) depict fine-scale movement colored by the concomitant ambient tag-recorded temperature

High-resolution, 2-min depth and temperature time-series records were obtained from recovered X-Tag 115974 (S20), providing fine-scale details on vertical behavior. The distribution of day and night depth records differed for this individual (Mann-Whitney test: *W* = 801,560,541, *P* < 2.2 × 10^−16^). The median daytime depth was 112.3 m (IQR = 231.6 m), and the median nighttime depth was 27.2 m (IQR = 67.9 m). Depth use showed clear variation between diel periods (Fig. 4). Although mostly associated with the epipelagic zone, this blue shark made repeated, directed excursions through the water column, reaching a maximum of 1030.8 m depth.

### Migratory movements

**Fig. 5:**
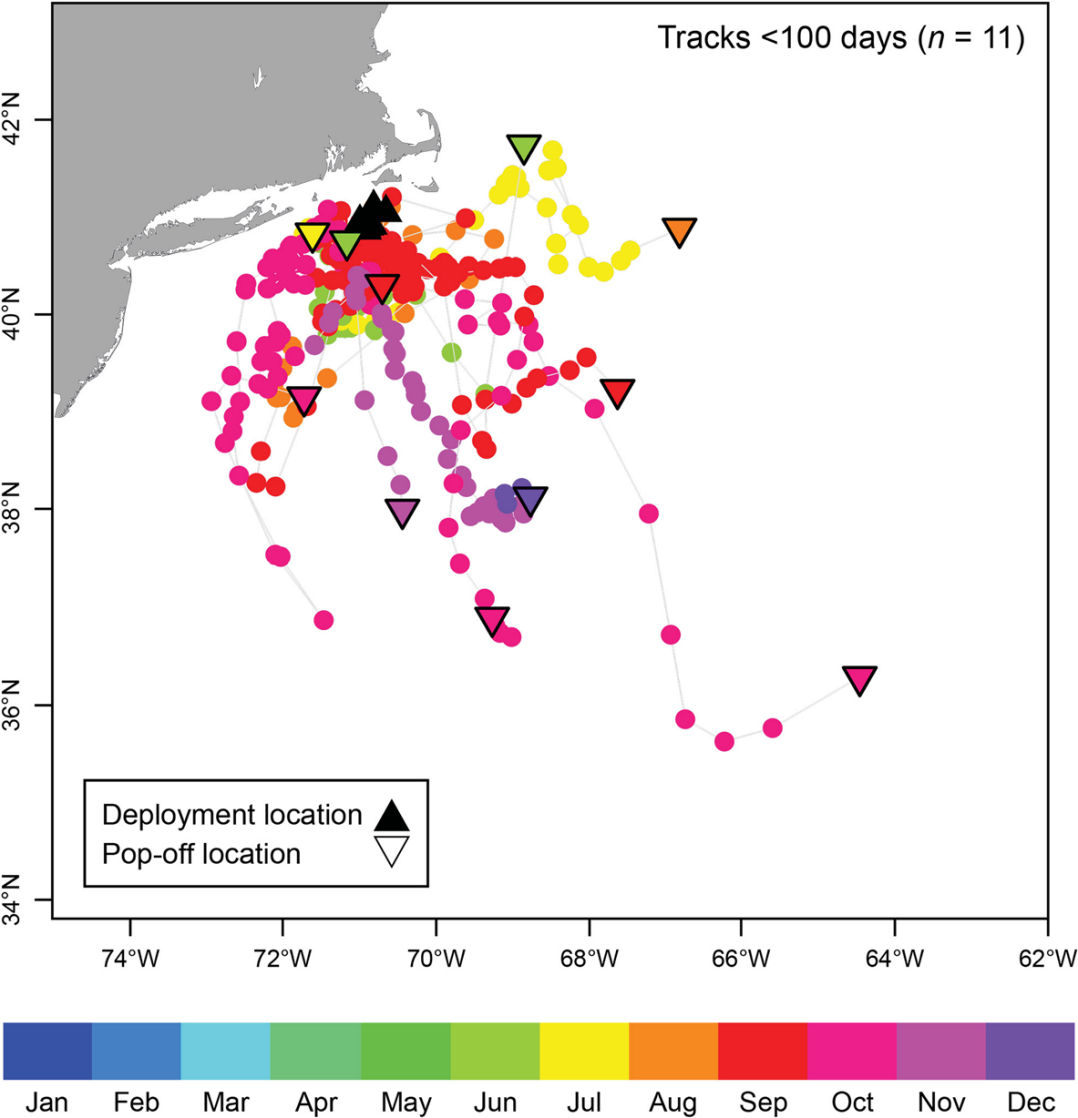
Short-duration (<100 days), horizontal movements for 11 individuals. Daily estimated locations are colored by month. Grey lines connect locations for each individual

For sharks tagged with SR tags (*n* = 18), tracking durations ranged from 6 to 273 days. From the UKFSST location estimates available, time spent in the United States Economic Exclusive Zone (EEZ) averaged 79.0 ± 33.0% time (range: 0–100% time). Three individuals entered or passed through The Bahamas’ EEZ. Individuals tracked for less than 100 days (*n* = 11), provided data from spring, summer, and fall seasons. Figure 5 illustrates short-duration migrations, resulting in net displacements ranging between 32.6 km and 767.8 km. Individual tracks tended towards the south and east of the tagging location.

**Fig. 6:**
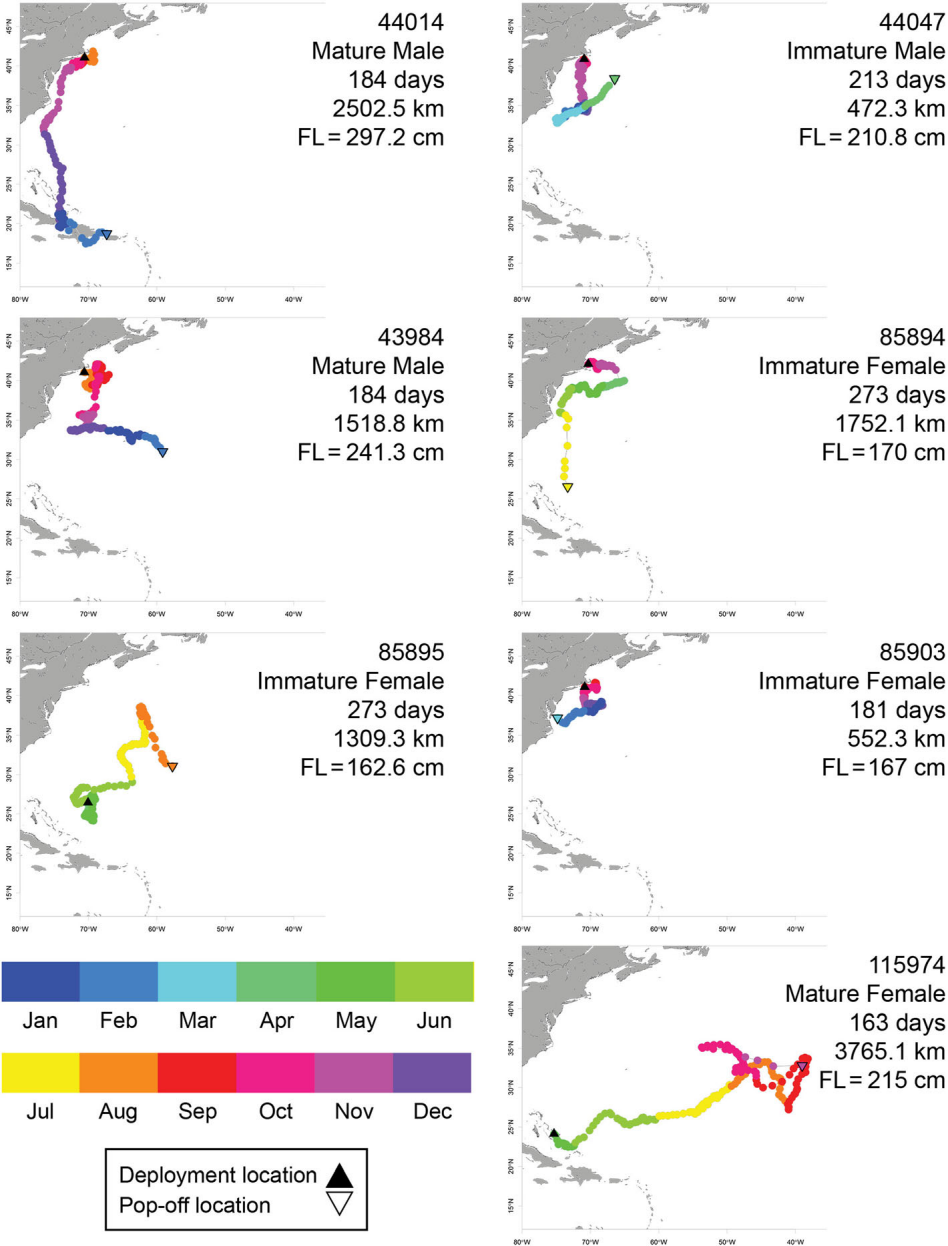
Long-duration (>100 days) horizontal movements for seven sharks. Colors correspond to individuals and daily estimated locations by month

**Fig. 7:**
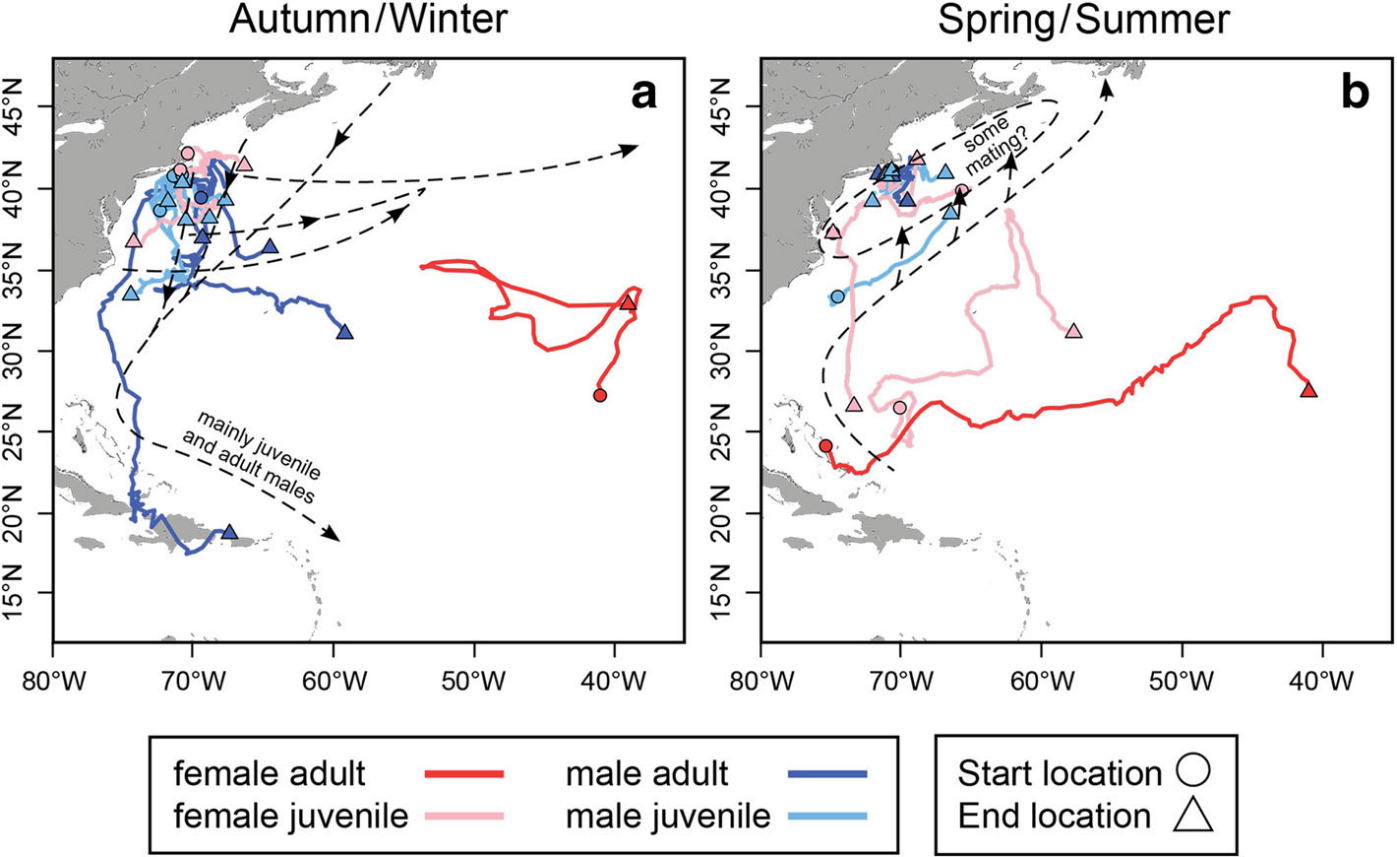
All horizontal movement tracks (*n* = 18) represented by solid colored line indicating demographic group and separated by season (**a**–**b**). Start and end locations represent the first and last locations within the corresponding season, respectively, for each individual. Existing/previously published/accepted western Atlantic blue shark migration model, previously inferred from mark-recapture data, is overlaid and represented by black dashed lines and text [25]

For all individuals tracked over 100 days (*n* = 7), long-range horizontal movements were recorded, resulting in net displacements ranging between 472.3 km and 3704.1 km (Figs. 6 and 7). Five individuals (44014, S12; 44047, S13; 43984, S8; 85894, S15; and 85903, S19) in this long-duration subset were tracked during winter months (December–February). All five were tagged on the continental shelf and all migrated away from the shelf in a south or southeast direction in October or November. While migrating, four of these individuals mostly occupied waters >20 °C while the remaining individual (85903, S19) primarily used waters <20 °C (Figs. 6 and 7).

The remaining long-distance tracks (85895, S16 and 115974, S20) resulted from transmitters deployed in the spring on females in the southern North Atlantic (Figs. 6 and 7). Shark 85895 (S16), tracked from April to August, exhibited a net migration in a northeast direction and subsequently remained in this warm (SST >20 °C) area for the duration of tracked time, contrasting to observations of other individuals in this study. An additional mature female with obvious mating wounds (115974, S20) was tagged in The Bahamas in May 2015 (Fig. 1). This female began migrating within two weeks of tagging and was recaptured (32.8 °N, 38.6 °W) by a European longlining vessel, after 163 days at liberty. This represents the largest net displacement (3704.1 km) by a shark in this study (Figs. 6 and 7).

## Discussion

PSATs proved to be an effective methodology to collect depth, temperature, and geolocation estimates from blue sharks during both coastal and migratory life phases, augmenting the limited data available to test movement hypotheses based on mark-recapture results from North Atlantic stocks. In addition to seasonally abundant mature males and immature (both sexes) blue sharks at an aggregation in the North Atlantic, this project opportunistically studied two adult females with recent mating wounds in the Caribbean (Fig. 1), which provided insight into mature female migration.

### On-shelf habitat use and description of the Northwest Atlantic aggregation

Although blue sharks are most commonly associated with oceanic waters and occur at depths between 0 and 1160 m [64], sharks in this study exhibited high site fidelity during summer and fall months to an area encompassing 391–2158 km^2^ on the shallow, neritic continental shelf of the northeastern U.S. While aggregating, blue sharks exhibited no significant depth differences based on presence in warm waters, between demographic groups, or diel period, suggesting shelf occupation is linked to a biological requirement of all demographics.

The blue sharks tracked in this study primarily used highly productive areas with chlorophyll *a* concentrations >1 mg/m^3^ and demonstrated lower rates of movement while aggregating than during migration, suggesting that primary productivity encourages site fidelity. The use of a distance metric in focal areas as a proxy for understanding daily dispersal of sharks would be beneficial in future studies. High primary productivity along with summer and fall elevations in species richness and food availability is well documented in the Narragansett Bay and Long Island Sound and coincided with summertime movements of blue sharks in this study [19, 64–66]. Comprehensive stomach content analysis of specimens collected in this area report that seasonal diet consists of cephalopods and a wide variety of teleosts, including swordfish (*Xiphias gladius*) and sea ravens (*Hemitripteridae* sp.) [32], species known to exhibit distinct diel-vertical habitat use. If blue sharks, indiscriminate of demographic group, aggregate on the continental shelf to exploit prey resources, they will exhibit overlap with the vertical habitat of their prey. Swordfish have surface-orientated nighttime basking behavior, potentially associated with periods of extended digestion and recovery from foraging events, which possibly makes the teleost vulnerable to predators, and loosely aligns with blue shark depths observed in this study [67]. Cephalopods exhibit strong patterns of diel vertical migration [68], and perhaps allows blue sharks to feed at night during periods of overlapping depth use. High seasonal productivity and localized abundance of blue shark prey items potentially explains the summer/fall aggregation of blue sharks, if sharks are capitalizing on prey abundance to gain energy reserves before undergoing energetically expensive long-distance migrations, similar to basking sharks (*Cetorhinus maximus*) [69].

Seasonal aggregations of sharks are often attributed to behaviors or processes related to reproduction [70]. Previous studies of the north Atlantic blue shark aggregation have hypothesized that mating scars on subadult and immature females suggest that some mating, and subsequent storing of sperm, occurs opportunistically during this seasonal aggregation [22, 25, 71]. However, the absence of mature females reduces the likelihood that mating is the primary purpose of this aggregation. We further hypothesize that absent mature females are already pregnant and avoiding unnecessary exposure to aggressive copulation. If shelf occupation fulfills a reproductive role, vertical niche partitioning between demographics would be evident because it limits energetically expensive mating to periods of overlapping habitat use [72]. Yet, males and immature females tracked during this aggregation exhibited corresponding depth use but discrete temperature use, alluding to fine-scale distinctions in horizontal distribution of demographics, not captured with light-based locations obtained by PSATs. Ambient temperature is a physiological regulator in all ectothermic sharks. Females, however, occupied cooler temperatures than mature and immature males on the shelf suggesting temperature has sex-specific biological significance in this species. Female blue shark skin is regularly thicker than male skin, presumably evolving from aggressive mating strategies [33], but perhaps also allowing for occupation of cooler temperatures, and ultimately, an expanded fundamental niche. Therefore, horizontal separation by females lowers the possibility of encountering an unneeded mate without affecting the likelihood of encountering blue shark prey items that exhibit ubiquitous distribution and consistent diel vertical migrations, such as cephalopods.

### Off-shelf depth and temperature use

All blue sharks demonstrated a change in depth use when embarking on fall migrations, displaying an expanded vertical niche and frequently diving below the thermocline. As sharks entered deeper waters, diel differences in depth and temperature use became evident. The biological significance of diurnal habitat use is unclear, since we cannot distinguish whether sharks select deeper water or cooler temperatures, because these variables are inherently associated. Carey and Scharold [37] suggested increased frequency and depth of blue shark dives represent hunting tactics employed by individuals searching for olfactory cues in the horizontally stratified ocean. Diel-depth differences may, therefore, be functionally associated with foraging [64]. Thus, switching prey types or decreased availability of prey off the shelf could explain these behaviors. However, prey types and foraging tactics of migratory blue sharks remain unknown. Ectothermic sharks typically seek physiologically optimal temperatures [37]. In this study, expanded vertical range off the continental shelf and the added effect of the Gulf Stream (SST >20 °C) resulted in deeper mean depths during migration. This indicates that the Gulf Stream is associated with blue shark vertical behavior only in the open ocean, off the shelf. This suggests that bathymetric availability primarily determines habitat use while temperature is a secondary determinant (as the Gulf Stream was not related to blue shark vertical behavior on the continental shelf). Of the five tracked individuals providing evidence of overwintering in lower latitudes, an immature female (85903, S20) and immature male (44047, S13) both migrated to the same approximate area east of South Carolina, U.S. However, the immature male consistently used comparably warmer waters (SST >20 °C) than the immature female (SST < 20 °C). Considering the expansive geographic range, which inherently includes a wide temperature range, and the directed selection of certain temperatures, particularly by males, it is unlikely that blue shark seasonal migrations are solely motivated by temperature-related physiological constraints. While foraging and thermoregulation may influence expanded depth use off the shelf, these hypotheses are confounded by the added effect of simultaneously undergoing migration.

Though the mechanisms of migration are poorly understood in pelagic animals, it is reasonable to assume that blue sharks must receive and interpret navigational cues during migrations covering thousands of kilometers. One hypothesis suggests that migratory sharks, such as scalloped hammerheads (*Sphyrna lewini*), dive below the thermocline to ascertain magnetic cues necessary to navigate [39, 73–75]. High-resolution data collected from the mature female contained dives into the mesopelagic zone during its 3765 km migration, suggesting deep, directed dives may be related to long-distance movements [76]. Additionally, blue sharks may assess bathymetric formations or detect undersea landmarks during dives, particularly the continental shelf ledge, potentially relevant to north-south movements [37]. If bathymetric cues are causative factors of depth selection during migration, sharks would exhibit greater depth use during periods of directed long-distance movement. In addition to navigational cues, the functionality of deep dives may fully, or in part, pertain to a number of alternative behaviors, such as predator avoidance, conspecific interactive behavior, or following prey [70, 76–78]. Distinct hypothesis testing of the biological significance of deep diving was beyond the scope of this study, and continues to challenge scientists, despite recent advances of tracking technologies [76]. Unlike the mature males in this study, immature males remained in the northwest Atlantic, not undergoing long-distance movements, and consistently selected significantly shallower depths, indicating that net displacement is related to deeper depths used by migratory sharks.

### Long-distance movements and comparison to Atlantic migration models

All blue sharks tagged in the northwest Atlantic moved southeast off the continental shelf (Fig. 7) [15, 25, 37]. Females did not exhibit mating wounds, however, if mating is closely followed by migration away from the shelf aggregation, it is possible that these females had not yet mated at the time of capture. One immature female traveled south to the tropics, and its tag reported from an area north of The Bahamas in July. The other immature female was tagged in southern waters in the spring, but made a net northeast migration to approximately 1600 km from the continental shelf aggregation in August. This further suggests that females may not return to the continental shelf in the spring and summer. Contrastingly, Atlantic blue shark movement hypotheses suggest males undergo northern migrations in the spring and annually return for summer residence on the continental shelf [22, 25]. An immature male (44047, S13), migrated northeast during March and April, and its tag released less than 500 km from the continental shelf aggregation, which supports annual philopatry to the shelf aggregation by males (Fig. 7). Remaining long-distance tracks concluded in February, prohibiting the assessment of springtime movements. However, current movement models hypothesize that northern movements do not commence until after February, which potentially explains the absence observed in our data [22]. Results from this study align with current blue shark migration hypotheses; however, both are inherently biased towards locations with large fishing efforts (i.e., northwest Atlantic). As such, future studies should aim to address deficiencies in geographical coverage.

Atlantic blue shark migration theory is especially deficient in describing mating areas or overlapping habitat for mature males and females. A single stock and an unstructured gene pool (authors’ unpublished data) suggest an overlapping spatial area characterized by mixing of adults. This, and other studies, have observed that some mature blue sharks migrate south to the Caribbean in the winter and spring [15, 36, 77, 78]. Additionally, all immature male sharks tracked in this study remained in the North Atlantic, not undergoing long-distance migrations, further supporting the hypothesis that mature blue sharks migrate to lower latitudes for reproductive purposes. Though unobserved at the New England study site, two adult female blue sharks with fresh mating wounds (personal observation ≤2 weeks) were captured discretely in The Bahamas. One female, migrated approximately 3704.1 km to the Mid-Atlantic Ridge, roughly 600 km southwest of the Azores. Mark-recapture data from the western Atlantic proves that a small percentage of tagged individuals make transatlantic migrations to the Azores, coasts of Portugal and Spain, Canary Islands, African coast, and Cape Verde Islands [22]. Sightings of neonate blue sharks and mature females in advanced stages of pregnancy suggest Azorean waters may serve as a springtime parturition area [36]. However, the spatio-temporal gaps in mark-recapture data allow the potential that some portion of the population uses Caribbean waters as a stopover or interim area before crossing the Atlantic to pupping grounds. If fresh mating wounds indicate pregnancy, it is possible the aforementioned female captured in The Bahamas was migrating to the eastern Atlantic adult female aggregation near the Azores [25]. This timeline suggests mature females may spend only several spring months in the Caribbean before departing to birthing areas and highlights the need to further examine the Caribbean as a potential mating area for North Atlantic blue sharks.

## Conclusions

Observations of blue sharks in this study highlight several ecology- and conservation-related issues important to resource managers. Blue shark vertical behavior varied greatly among locales, and sexual segregation and seasonal site-faithfulness were evident from tracking data. Most noticeably, blue sharks aggregated on the continental shelf used cool temperatures (SST < 20 °C), shallow depths (mean maximum daily depth 45.9 ± 25.8 m), and were possibly horizontally clustered by demographic group. As a consequence, densely aggregated (vertically and horizontally) juveniles and reproductive subadults are incidentally targeted when recreational and commercial fisheries are intensive in this region and could potentially affect Atlantic-wide populations of blue sharks [27]. High primary productivity and prey availability, absence of mature females, and distinct temperature use by immature females, suggests nutrient intake is the primary physiological need fulfilled in seasonally aggregating sharks on the northeast Atlantic continental shelf. Therefore, ecosystem and food web dynamics should be incorporated into management plans pertaining to this U.S. jurisdiction.

Seven tags were retained by individuals over multiple seasons and corresponded to existing Atlantic migration models. Highly migratory animals that navigate through international jurisdictions require multinational cooperative conservation plans, particularly sexually segregated animals such as blue sharks. For this level of management, detailed information regarding migration routes and movement ecology are necessary. Though absent from decades (and >100,000 blue sharks) of mark-recapture data collection, this needed resolution of data is now acquirable through satellite tagging surveillance. Three sharks entered the Bahamian EEZ, the only encountered jurisdiction in this study that offers protection to sharks. However, given the probable connectivity of western and eastern Atlantic blue shark populations, a single refuge is not sufficient to protect this highly mobile species, particularly if the Caribbean harbors a mating area as suggested by the recently mated females observed during this study. Further support for Atlantic-wide management is underscored by the long-distant movement and subsequent capture by a commercial fishery of one individual (S20) in a hypothesized pupping area [25, 36].

Wide-ranging migrations and sexually segregated seasonal aggregations highlight the complexity of blue shark life history [21], and satellite tagging studies, often limited in sample size by research cost, have focused tagging efforts in one location, inadvertently targeting a distinct demographic from the population [15, 26, 64]. Quantifying the demographic-specific relationship between the environment and elicited behavioral response is integral in determining the motive(s) driving northwest Atlantic aggregations and migrations. Our results align with previous reports that blue sharks demonstrate variable, ocean-wide migrations, and adapt to differing habitats by modifying vertical behavior [15, 64]. However, demographic distinctions revealed by this study suggest that habitat is selected in response to physiological requirements and warrants further directed study which considers the added effects of (a) biotic factors. The ongoing need to supplement deficits in current movement models coupled with the complex interplay of physiological requirements and restrictions, environmental variables, resource availabilities, and reproductive needs that dictate blue shark ecology supports the need for a collaborative, multilateral approach to future research studies and management plans.

## Additional file

Additional file 1: Details regarding the models constructed to assess the effect of the continental shelf, Gulf Stream, period of day, and demographic group on the depth use of blue sharks in this study. (DOCX 13 kb)

## Abbreviations

EEZ: Exclusive Economic Zone
HR: High Rate
PSAT: Pop-up satellite archival tag
SR: Standard Rate
SST: Sea surface temperature
UKFSST: Unscented Kalman filter with sea surface temperature
