# Supplementary material for: Biogeophysical and physiological processes drive movement patterns in a marine predator
Source: Mov Ecol. 2017 Jul 18;5:16. doi: 10.1186/s40462-017-0107-z (PMC5514520; doi:10.1186/s40462-017-0107-z)
Supplement: Additional file 1: — Details regarding the models constructed to assess the effect of the continental shelf, Gulf Stream, period of day, and demographic group on the depth use of blue sharks in this study. (DOCX 13 kb) [file 40462_2017_107_MOESM1_ESM.docx]

**Additional file 1**

In all model equations, let $i$ correspond to the $i^{th}$shark and $j$ correspond to the $j^{th}$ observation within the $i^{th}$shark.

In the first linear mixed effects model, we consider the main effects of the continental shelf, marked by the 200 m bathymetry contour, and the Gulf Stream, marked by the 20°C sea-surface temperature threshold.

$$y_{ij}=\beta_{0}+\beta_{1}x_{ij1}+\beta_{2}x_{ij2}+\beta_{12}x_{ij1}x_{ij2}+\delta_{i}+\varepsilon_{ij}$$

Here $y_{ij}$ corresponds to the daily mean depth response variable and $\beta_{0}$ corresponds to the intercept. Presence on the continental shelf and in the Gulf Stream are represented by indicator variables $x_{ij1}$ and $x_{ij2}$, respectively defined as follows:

$$x_{1}=\left\{ \begin{matrix} 0 & on shelf \\ 1 & off shelf \end{matrix} \right.$$

$$x_{2}=\left\{ \begin{matrix} 0 & in Gulf Stream \\ 1 & out of Gulf Stream \end{matrix} \right.$$

It follows that $\beta_{1}$, $\beta_{2}$, and $\beta_{12}$ represent the regression coefficients of the corresponding regressors and their interaction. The random effect for each shark is modeled by the $\delta_{i}$ term, and the error within each shark is represented by the $\varepsilon_{ij}$ term.

The four subsequent models are evaluated on the data partitioned by habitat, either on or off the continental shelf. Although the models assess different response variables (depth and temperature) in differing habitats (on and off the continental shelf), the model structure remains consistent and is displayed as follows:

$$y_{ij}=\beta_{0}+\beta_{1}x_{ij1}+\beta_{2}x_{ij2}+\beta_{3}x_{i3}+\beta_{4}x_{i4}+\delta_{i}+\varepsilon_{ij}.$$

Here $y_{ij}$ corresponds to either the daily day/night mean depth or temperature. The model assesses the effects of diel period (day or night), demographic group (mature male, immature male, and immature female), and presence in the Gulf Stream (in or out) by modeling these effects as indicator variables defined as follows:

$$x_{1}=\left\{ \begin{matrix} 0 & on shelf \\ 1 & off shelf \end{matrix} \right.$$

$$x_{2}=\left\{ \begin{matrix} 0 & in Gulf Stream \\ 1 & out of Gulf Stream \end{matrix} \right.$$

$$x_{3}=\left\{ \begin{matrix} 0 & immature female \\ 1 & immature male \\ 0 & mature male \end{matrix} \right.$$

$$x_{4}=\left\{ \begin{matrix} 0 & immature female \\ 0 & immature male \\ 1 & mature male \end{matrix} \right.$$

Therefore, $\beta_{1}$, $\beta_{2}$, $\beta_{3},$ and $\beta_{4}$ represent the regression coefficients of the corresponding regressors, and $\beta_{0}$ represents the intercept. The random effect for each shark is modeled by the $\delta_{i}$ term, and the error within each shark is represented by $\varepsilon_{ij}$ term.
